# Methoxyamine Enhances 5-Fluorouracil-Induced Radiosensitization in Colon Cancer Cell Line HT29

**DOI:** 10.22074/cellj.2016.4295

**Published:** 2017-02-22

**Authors:** Samideh Khoei, Roghayeh Poorabdollahi, Ahmad Mostaar, Fariborz Faeghi

**Affiliations:** 1 Razi Drug Research Centre, School of Medicine, Iran University of Medical Sciences, Tehran, Iran; 2 Department of Medical Physics, School of Medicine, Iran University of Medical Sciences, Tehran, Iran; 3 Department of Radiology Technology, School of Allied Medical Sciences, Shahid Beheshti University of Medical Sciences, Tehran, Iran; 4 Department of Medical Physics and Medical Engineering, School of Medicine, Shahid Beheshti University of Medical Sciences, Tehran, Iran

**Keywords:** 5-Fluorouracil, Methoxyamine, Radiation, Comet Assay, Colon Cancer

## Abstract

**Objective:**

This study intended to observe the effects of methoxyamine (Mx) on cytotoxic effects and DNA damage caused by 5-Fluorouracil (5-FU) in combination with gamma radiation in a human colon cancer cell line, HT29.

**Materials and Methods:**

In this experimental study, HT29 cells were cultured as a monolayer and treated with different concentrations of 5-FU along with 1 mM Mx for 24 hours. Next, the cells were irradiated with 2 Gy gamma radiation. After the treatments, we assessed for DNA damage, cytotoxicity, and viability by alkaline comet, clonogenic survival, and trypan blue dye exclusion assays.

**Results:**

Cytotoxicity and DNA damage increased with increasing 5-FU concentration. The 1 mM Mx concentration had no significant effect on cytotoxicity and DNA damage from 5-FU; however, it increased the cytotoxic and genotoxic effects of different concentrations of 5-FU when used in combination with 2 Gy gamma radiation.

**Conclusion:**

Mx combined with 5-FU enhanced the radiosensitivity of colon cancer cells.

## Introduction

Colorectal cancer is considered the third most prevalent cancer worldwide. In 2013, an estimated 102,480 new cases and 50830 deaths by colon and rectal cancers were reported in the United Stated-a rate of 40 out of 100,000 humans annually (1,2). Surgery, radiotherapy, and chemotherapy are currently the main modalities to treat colon cancer. However, damage to healthy, normal tissue is considered the most important obstacle against radiation therapy (3). In recent years, combined chemo- and radiation therapy comprised the adjuvant treatment for the majority of cancer cases (4). 5-fluorouracil (5-FU), one of the most important, widely used chemotherapeutic agents for colorectal cancer, was first introduced as an anti-metabolite in 1957 (5). 5-FU is a halogenated pyrimidine that converts to several active metabolites with various mechanisms of action, including inhibition of thymidylate synthase (TS) by 5-fluoro-2'-deoxyuridine-5'-monophosphate (FUdMP). The insertion of 5-fluorouridine- 5'-triphosphate (FUTP) into RNA, as well as the insertion of 5-fluoro-2'-deoxyuridine- 5'-triphosphate (FdUTp) into DNA has been reported (6). However, 5-FU either directly or indirectly mediates cytotoxicity by interference of RNA and DNA functions (7). Geng et al. (8) have shown that 5-FU incorporation into the genome is also recognized by two DNA repair pathways that may play a role in the survival of cells treated with 5-FU; one pathway is the base excision repair (BER), while the other repair pathway is the mismatching repair (MMR) system. BER is one of the major DNA repair pathways for 5-FU, but 5-FU cytotoxicity depends mainly on insertion in RNA (9). BER is a cell repair pathway to remove DNA damages such as single strand breaks (SSBs), double strand breaks (DSBs), and base damages (10). Methoxyamine (Mx) is a specific chemical inhibitor of BER that acts by tightly binding to the Apurinic/apyrimidinic (AP) site generated by the cleavage of BER glycolysis which renders the phosphodiester bond agent at the AP site refractory to the catalytic activity of AP endonuclease (11). Chemical inhibition of BER by Mx is a valid pharmacologic strategy to potentiate the cytotoxicity of chemotherapeutic agents such as temozolomide (12), 5-Iodo-2’-deoxyuridine (IUdR) (13), and 5-Fluorodeoxyuridine (FUdR) (14). However, Pettersen et al. (9) have revealed that Mx contributed negligibly to FU cytotoxicity, which was reversed by the incorporation of RNA. The present study evaluated the effects of Mx on 5-FU radiosensitivity in the presence of ionizing radiation in the HT29 colon cancer cell line. 

## Materials and Methods

This experimental study performed on the HT29 human colon cancer cell line received the approval of the Ethical Committee of Iran University of Medical Sciences (code number: 92-01-20204). 

### Cell line

The human colorectal cancer cell line, HT29 (Pasteur Institute, Iran), was cultured in RPMI- 1640 (PAA Laboratories GmbH, Austria) media supplemented with 10% fetal bovine serum (FBS, PAA Laboratories GmbH, Austria), 100 U/ml of penicillin, and 100 mg/ml of streptomycin (PAA Laboratories GmbH, Austria). 

### Monolayer culture

Cells were cultured as a monolayer at a density of 104 cells/cm^2^ in T-25 tissue culture flasks (Orange Scientific). Cultures were maintained at 37˚C in a humidified atmosphere and 5% CO_2_. Cells were detached with 0.25% trypsin and 0.03% ethylenediaminetetraacetic acid (EDTA, Sigma, USA) in phosphate-buffered saline (PBS, Sigma, USA). 

### Trypan blue dye exclusion assay

HT29 cells were cultured at a density of 2×10^4^ per well in multiwell plates (24 wells/plate, SPL). After 24 hours, we treated the cells with different concentrations of 5-FU (0, 1, 5, 10, 50, 100 µM) or Mx (0, 1, 6, 30, 60, 120 mM). After 24 hours, cell viability was determined by the trypan blue dye exclusion assay. We considered viability as the percentage of unstained cells out of total number of cells for each cell category. 

### Colony formation assay

"The colony formation assay is an *in vitro* cell survival assay based on the ability of a single cell to grow into a colony" (15) . In this assay, we treated the cells with different concentrations of 5-FU (0, 0.01, 0.1, 1 and 10 µM) or Mx (0, 0.01, 0.1, 1 and 6 mM). After 24 hours, single cell suspensions were seeded onto 60-mm petri dishes (Orange Scientific, Braine l’Alleud, Belgium) and grown in RPMI that contained 10% FBS. The cells were incubated at 37˚C in a humidified atmosphere of 5% CO_2_for 10 days. After this interval, the colonies which contained a minimum of 50 cells were counted by an inverted phase microscope and we used the following equation to calculate the plating efficiency: 

Plating efficiency =(Number of colonies counted)/ (Number of cells plated) ×100

###  Irradiation procedure 

For gamma radiation, we replaced the medium with fresh medium. The cells were irradiated using a^60^Co source (Theratron-780c, MDS Nordion) at a dose rate of 87 cGy/minute for 2 Gy. We conducted the radiation treatment by placing the culture flasks under collimator of equipment at an 80.5 cm distance between the head of the device and the floor of the flasks. The field size was 20×15 cm^2^ for a 2.30 minute irradiation period. 

### Cell treatment 

A total of 5×10^5^ HT29 cells were seeded in a T25 culture flask (SPL). After 24 hours, cells received 5-FU, Mx and 2 Gy of gamma radiation based on the following 8 groups. The cells that received 5-FU and/or Mx were treated for 24 hours at 37˚C in a humidified atmosphere and 5% CO_2_. Treatments were performed according to the following 8 groups:

1 Control without treatment
2 Mx (1 mM) for 24 hours
3 5-FU at 0.1, 1, or 5 μM for 24 hours
4 Gamma radiation (2 Gy)
5 Simultaneous Mx (1 mM) plus 5-FU (0.1, 1, or 5 μM) for 24 hours
6 Mx (1 mM) for 24 hours followed by gamma radiation (2 Gy)
7 5-FU (0.1, 1, or 5 μM) for 24 hours followed by gamma radiation (2 Gy)
8 Simultaneous Mx (1 mM) plus 5-FU (0.1, 1, or 5 μM) for 24 hours followed by gamma radiation (2 Gy).

The viability of control and treated cells were determined by the trypan blue dye exclusion assay. Cytotoxic effects and DNA damages were measured according to the clonogenic and alkaline comet assays. 

### Alkaline comet assay

The comet assay or single-cell gel electrophoresis (SCGE) is a standard method for assessment of DNA damage. We have assessed the presence of DNA fragmentation according to the Comet assay (16). Fluorescence intensity was measured using CometScore software (TriTek Corp., Sumerduck, VA) and reported as the Comet tail moment. 

### Statistical analysis

Data were reported as mean ± SEM with n denoting the number of experiments. We used one-way analysis of variance (ANOVA) followed by Tukey’s test as the post hoc with SPSS version 16 for data analysis. P<0.05 was considered to be statistically significant. 

## Results

### Cell characteristics

The HT29 colon cancer cell line grew as a monolayer on the tissue culture flasks with a population doubling time calculated from the growth curve of 22.5 ± 0.13 hours. 

### Effects of 5-Fluorouracil on viability and clo- nogenic ability of HT29 cells

**Fig.1:**
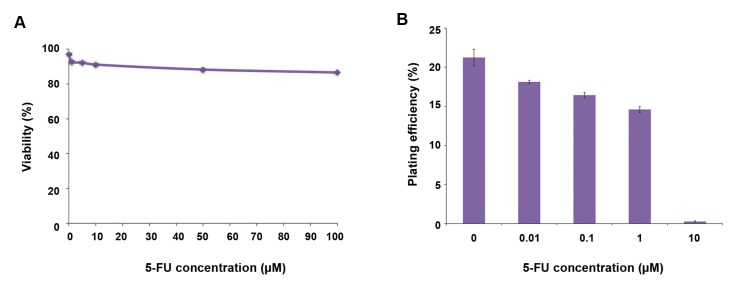
The effect of 5-Fluorouracil on the viability and clonogenicity of HT29 colon cacncer cell line. A. Viability of HT29 cell line after a 24-hour incubation period with 0-100 µM of 5-Fluorouracil (5-FU) (All concentrations vs. control P<0.005) and B. Clonogenic ability of the cell after the 24-hour incubation period with 0-10 µM of 5-FU (0.1 and 1 µM concentrations vs. control: P<0.05 and 10 µM vs. control: P<0.005). Mean ± SEM of 3 experiments.

We treated the cells that had been cultured for 24 hours in a T25 culture flask with different concentrations of 5-FU. After 24 hours of treatment, we assessed cell viability and colony formation ability by the trypan blue dye exclusion and colony formation assays. Figure 1A and B show the effects of 5-FU on viability and clonogenic ability of HT29 cells. Viability decreased from 96 to 86% when the concentration of 5-FU increased from 0 to 100 μM. All concentrations from 1 to 100 μM significantly affected cell viability compared to the control cells (P<0.005, Fig .1A). We conducted the clonogenic assay on cells treated with 0 to 10 μM 5-FU. Cells treated at these concentrations had more than 90% viability. The colony formation ability of the cells significantly decreased with the higher 5-FU concentrations of 0.1 and 1 μM versus the control (P<0.05) and 10 μM 5-FU versus the control (P<0.005, Fig .1B).

### Effects of methoxyamine on viability and clono- genic ability of HT29 cells

**Fig.2:**
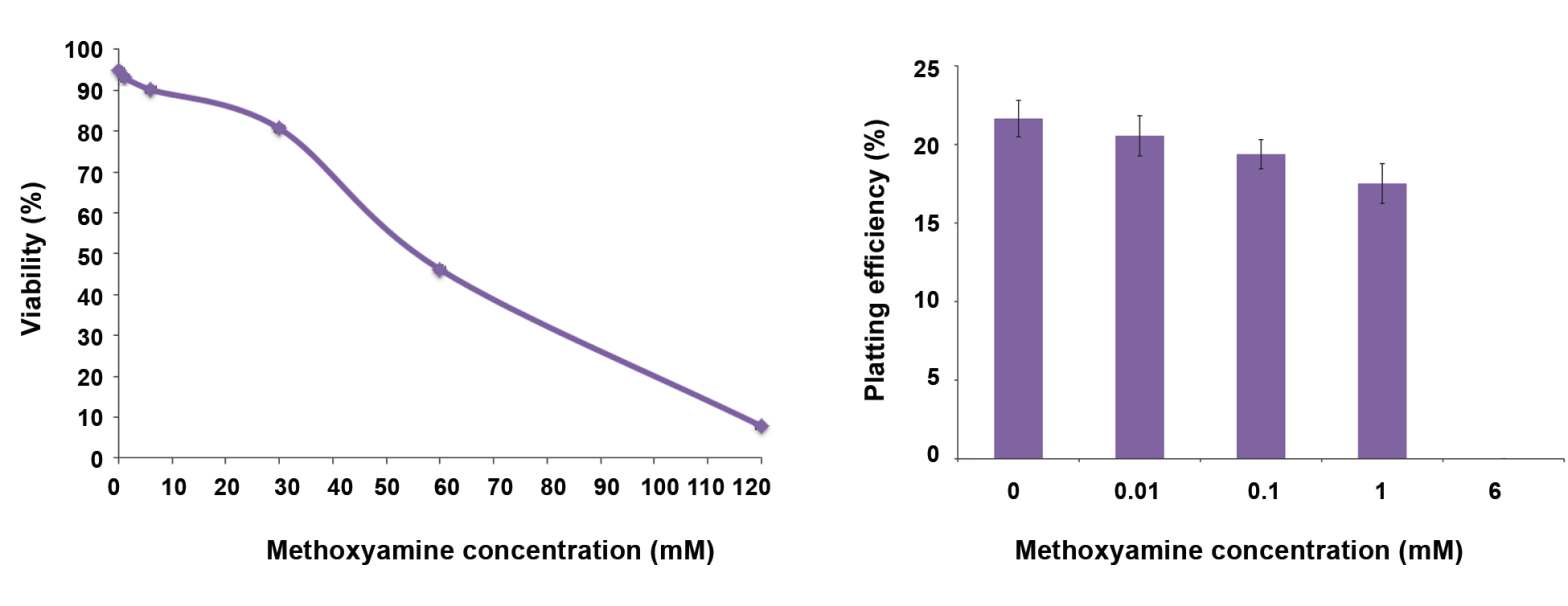
The effect of methoxyamine on the viability and clonogenicity of HT29 colon cacncer cell line. A. Viability of the HT29 cell line after a 24-hour incubation with 0-120 mM of methoxyamine (Mx) (6 mM vs. control: P<0.005 and 30 to 120 mM vs. control: P<0.001), B. Clonogenic ability of the cell after a 24-hour incubation with 0-6 mM of Mx. (0.01, 0.1 and 1 vs. control: P>0.05, 6 mM vs. control: P<0.001). Mean ± SEM of 3 experiments.

We treated the cells with different concentrations of Mx after 24 hours of culture. Viability and colony formation ability of the cells were assayed with the trypan blue dye exclusion and colony formation assays at 24 hours after treatment. Figure 2A and B show the effect of Mx on viability and clonogenic ability of the HT29 cells. Viability significantly decreased from 95 to 5% when the concentration of Mx increased from 6 mM versus the control (P<0.005) and from 30 to 120 mM versus the control (P<0.001, Fig .2A). The clonogenic assay was performed on cells treated with 0-6 mM of Mx where the viability of treated cells was more than 90%. Different concentrations of Mx up to 6 mM (0.01, 0.1 and 1 mM) had no significant effect on colony formation ability of the cells (P>0.05), whereas 6 mM Mx significantly reduce the number of colony (P<0.001, Fig .2B). 

### Effects of methoxyamine, 5-Fluorouracil, and gamma radiation on cell viability 

**Fig.3:**
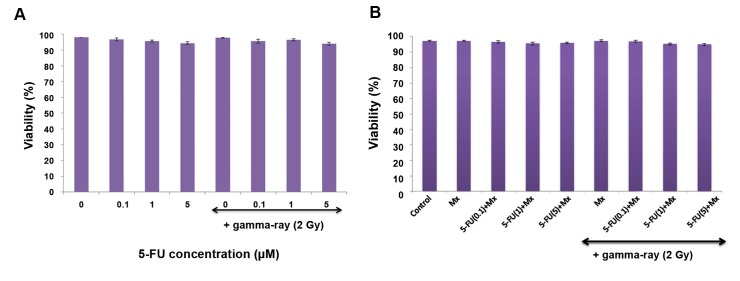
The viability of control and treated cell with 5-Fluorouracil alone or in combination with gamma radiation and methoxyamine. A. Effects of different concentrations of 5-Fluorouracil (5-FU) with/without gamma radiation (5-FU alone vs. 5-FU+gamma radiation: P˃0.05) and B. Different concentrations of 5-FU in combination with 1 mM methoxyamine (Mx) with/without gamma radiation on viability of HT29 cells in a monolayer culture. HT29 cells were treated for 24 hours with either 0, 1, 0.1, or 5 µM of 5-FU alone or in combination with 1 mM Mx, followed by 2 Gy gamma radiation. The viability was determined at the end of radiation exposure using the trypan blue dye exclusion assay (5-FU+1mM Mx vs. 5-FU+1mM Mx+gamma radiation: P˃0.05). Mean ± SEM of 3 experiments.

Immediately after cell treatment with Mx, 5-FU, and radiation, we counted the cells and determined viability by the trypan blue dye exclusion assay. Figure 3A and B show the effects of Mx, 5-FU, radiation, and their combination on the viability of HT29 cells. Different concentrations of 5-FU (0-5 µM) alone or in combination with 2 Gy gamma radiation had no significant effect on cell viability (P>0.05, Fig .3A). Figure 3B shows that different concentrations of 5-FU combined with Mx in the presence or absence of gamma radiation had no significant effect on cell viability (P>0.05). 

### Effects of methoxyamine, 5-Fluorouracil, and gamma radiation on colony forming ability

**Fig.4:**
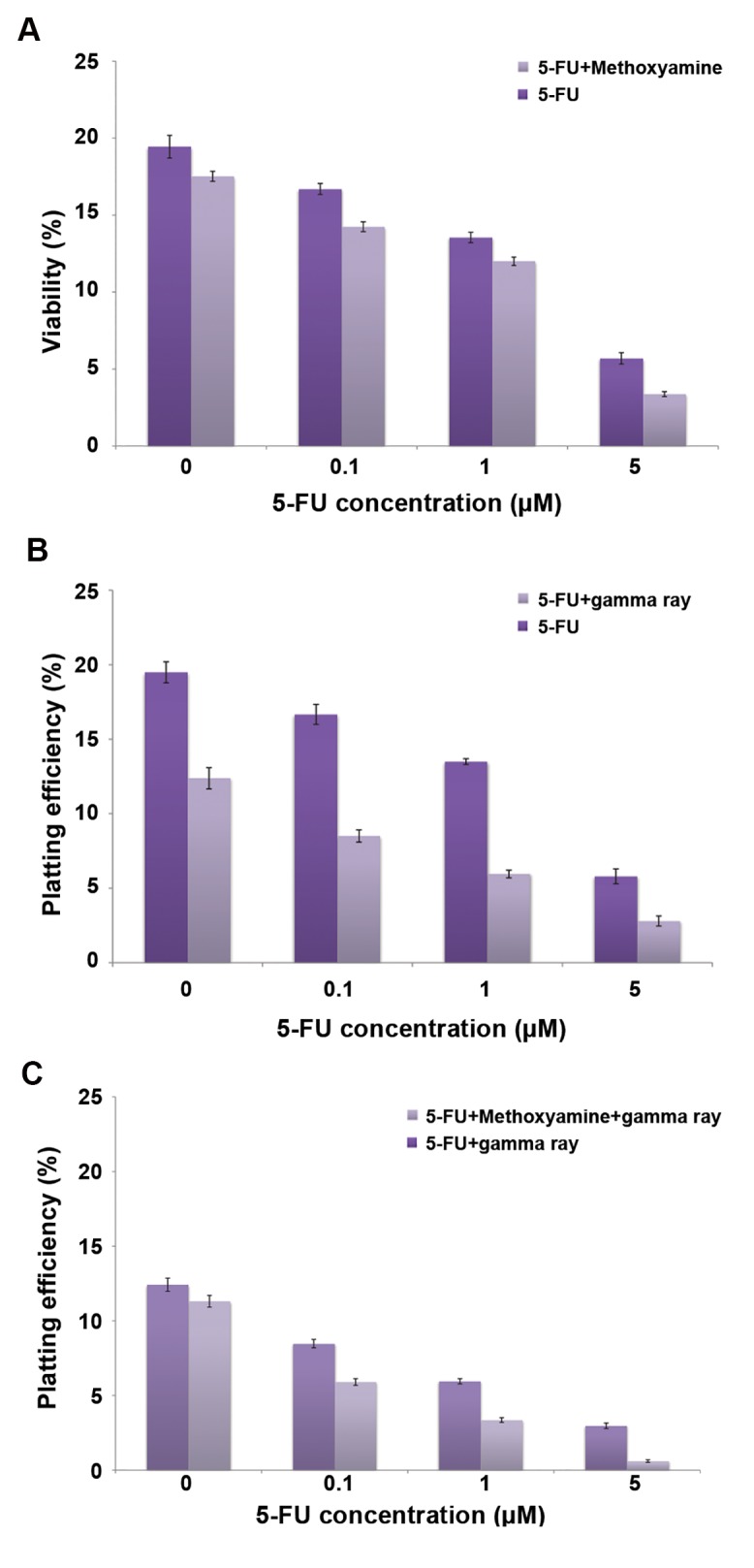
The colony formation ability of control and treated cell with 5-Fluorouracil alone or in combination with gamma radiation and methoxyamine. A. Plating efficiency of HT29 cells treated with different concentrations of 5-Fluorouracil (5-FU) alone or in combination with 1 mM methoxyamine (Mx). (5-FU alone versus 5-FU+1 mM Mx: P˃0.05), B. Different concentrations of 5-FU alone or in combination with gamma radiation (5-FU alone versus 5-FU+gamma radiation: P˂0.05; up to 1 µM 5-FU: P<0.001, 5 µM 5-FU: P=0.03), and C. Different concentrations of 5-FU in combination with gamma radiation with and without 1 mM Mx. HT29 cells were treated for 24 hours with 0, 1, 0.1, or 5 µM of 5-FU alone or in combination with 1 mM Mx, followed by 2 Gy gamma radiation. The colonies were counted 10 days after treatment. Mean ± SEM of 3 experiments. (5-FU+gamma radiation vs. 5-FU+gamma radiation+1 mM Mx; P<0.001).

We analyzed the cellular response to varying doses of 5-FU, 1 mM Mx, and 2 Gy radiation in terms of colony formation in 7 groups of samples. Plots of plating efficiency versus different treatments of HT29 cells are shown in Figure 4A-C. Figure 4A shows that the colony formation ability of the cells decreased with increased 5-FU concentrations in the 5-FU only group and 5-FU+1 mM Mx group. The 1 mM Mx dose had no significant effect on cytotoxicity at different concentrations of 5-FU (P>0.05). Figure 4B shows that the colony formation ability of the cells decreased along with increased 5-FU concentrations in the 5-FU only group and 5-FU+2 Gy gamma radiation group. However, the 5-FU+gamma radiation treatment caused a significant reduction in the number of colony forming cells compared with 5-FU alone at all concentrations of 5-FU (up to 1 µM: P<0.001, 5 µM: P=0.03). Figure 4C shows that colony formation ability of the cells decreased along with increased 5-FU concentrations in both groups: 5-FU+gamma radiation and 5-FU+gamma radiation+Mx. However, the number of colony forming cells significantly reduced in the 5-FU+gamma radiation+Mx group compared to the 5-FU+gamma radiation group at all concentrations of 5-FU (P<0.001). 

### Effects of methoxyamine, 5-Fluorouracil and gamma radiation on induced DNA damage

**Fig.5:**
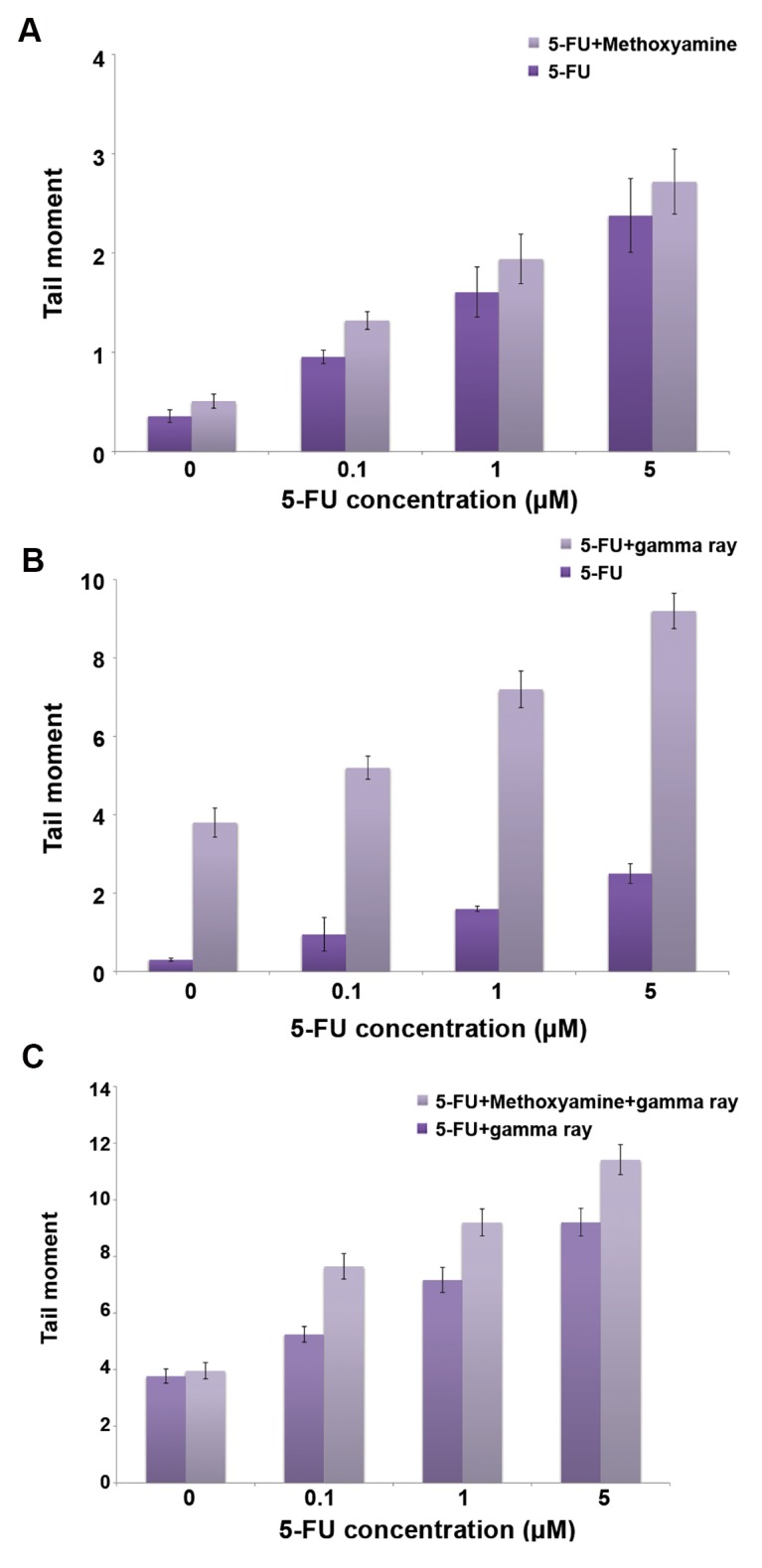
The level of DNA damages in control and treated cell with 5-Fluorouracil alone or in combination with gamma radiation and methoxyamine. A. Calculated tail moments after treatment with different concentrations of 5-Fluorouracil (5-FU) alone or in combination with 1 mM methoxyamine (Mx) (5-FU alone vs. 5-FU+1 mM Mx: P<0.05), B. Different concentrations of 5-FU alone or in combination with gamma radiation (5-FU alone vs. 5-FU+gamma radiation: P<0.001), and C. Different concentrations of 5-FU in combination with gamma radiation with/without 1 mM Mx. HT29 cells were treated for 24 hours with 0, 1, 0.1, or 5 µM 5-FU alone or in combination with 1 mM Mx, followed by 2 Gy gamma radiation. The comet assay was performed immediately after cell treatment. (5-FU+gamma radiation vs. 5-FU+gamma radiation+1 mM Mx; P<0.001). Mean ± SEM of 3 experiments.

Alkaline comet assays were used to evaluate DNA damage after drug treatment and ionization radiation. The average of the tail moments in the cells was used to show DNA damage of the cells. Plots of tail moment versus different treatments of HT29 cells are shown in Figure 5A-C. Figure 5A shows that DNA damage to the cells increased with increased 5-FU concentrations in both groups: 5-FU alone and 5-FU+1 mM Mx. However, the 1 mM Mx concentration had no significant effect on the level of DNA damage caused by different concentrations of 5-FU (P> 0.05). Figure 5B shows that DNA damage in the cells increased with an increase in 5-FU concentrations in both groups: 5-FU alone and 5-FU+2 Gy of gamma radiation. However, the extent of induction in the damage of DNA caused by 5-FU+gamma radiation significantly increased compared to 5-FU alone at all concentrations of 5-FU (P<0.001). Figure 5C shows that DNA damage to the cells increased with increased 5-FU concentrations in both groups: 5-FU+gamma radiation and 5-FU+gamma radiation+Mx. However, the extent of induction of DNA damage caused by 5-FU+gamma radiation+Mx significantly increased compared with 5-FU+gamma radiation at all concentrations of 5-FU (P<0.001). 

## Discussion

Colorectal cancer is the third cause of cancer death worldwide (2).This study has investigated the human colon cancer cell line HT29 in a monolayer culture model. Transmission of the maximum therapeutic dose of ionizing radiation to the considered volume of cancer tissues is the ultimate goal of radiotherapy (17). Today, most researchers consider combined treatments in order to reduce the radiation dose to normal tissue while increasing damage to tumors. 5-FU is one of the most commonly used chemotherapeutic agents for colorectal cancer. In addition, it has been used extensively with radiation. 5-FU is a metabolic analogue of thymine in DNA synthesis or uracil in RNA synthesis. Replacement of this analogue inhibits DNA synthesis in cells that actively divide. 5-FU can enhance the cytotoxicity of ionizing radiation (7). 

A number of cell repair pathways activate after cell damage, of which one of the most promising DNA repair pathways is BER (18). However, the chemical agent Mx can interfere with this repair pathway (19). Mx can prevent repair progression by forming sustainable bonds with areas in DNA without bases (20). Higher concentrations of 5-FU lead to decreases in the percentage of cell survival and cell proliferation because of increased drug toxicity as confirmed by Fan et al. (21) and Sasaki et al. (22). These researchers have investigated 5-FU cytotoxicity on the colon cancer cell line HT29 in a monolayer culture (MTT test). They observed that higher concentrations of 5-FU led to a decrease in the percentage of surviving cells and increased cell toxicity. We showed that higher concentrations of Mx increased cell toxicity and decreased cell proliferation ability. This result was similar to results obtained by Liu et al. (10) who injected different concentrations of Mx into nude mice and observed that higher concentrations caused the mice to die. 

Montaldi et al. (23) reported similar effects on osteosarcoma cells according to the MTT test. As seen striking differences exist in terms of clonogenic survival between concentrations 1 and 10 µM of 5-FU whereas there is no difference as measured with trypan blue. Same result seen between concentrations 1 and 6 mM of Mx. In this study, we used the trypan blue dye exclusion assay to determine cell viability immediately after treatment. 

However, we have determined the colony formation ability of the cells after 10 days with the clonogenic assay. Differences exist between the trypan blue dye exclusion assay and clonogenic assay. For example, it is possible that different types of cells may require different periods of time for a loss of membrane integrity to occur following a lethal injury. Some drugs may induce cell cycle delays not associated with cell death. Certain drugs may possibly cause a lethal injury which does not become manifest until several cell generations later. If colonies were counted after 6 generation times (the time required for a 64-cell colony to form), then a clone which died after 3 generation times would be detected in the clonogenic assay, but not detected if a nonclonogenic assay was performed within the first 3 generation times (24). The current study has shown that the number of colonies formed by cells in the group treated with different concentrations of 5-FU+Mx did not significantly different from those seen in the group treated with different concentrations of 5-FU alone, which was confirmed by the comet assay. 

Pettersen et al. (9) showed that a low concentration of Mx did not influence inhibition of the damage repair of a base incision due to 5-FU. Mx had no effect on cell proliferation and did not lead to DNA damage in the cells. They investigated the importance of the BER pathway in 5-FU toxicity by using the signaling inhibitors for DNA and BER damage. They observed that Mx did not influence 5-FU toxicity. Geng et al. (8) confirmed the accuracy of these findings. Mx did not influence DNA damage, nor did it decrease cell proliferation as observed in studies by Yan et al. (11). The most important reason to limit clinical administration of 5-FU after 50 years would be increased tolerance by tumor cells (25). These studies have shown that a synergistic effect may be obtained when 5-FU is used in combination with hyperthermia (26). The current study results indicated that increased concentrations of 5-FU could significantly decrease cell proliferation compared to the control group. However, the resultant tail moments in the control and 5-FU treated groups showed no significant difference at all concentrations, except for 5 µM. This difference between the comet and clonogenic assay results could be due to the evaluation time in these two assays. Colony formation ability was assayed after 10 days. Therefore, the cells had enough time to repair DNA and cell damage. The alkaline comet assay shows only SSB, DSB and AP sites. In this study, we assessed DNA damage immediately after treatment. Therefore, in contrast to the colony formation assay, these cells did not have enough time to repair the DNA damage. As a result, all damage could be detected using the alkaline and neutral comet assay. Hajikarimi et al. (27) and Mohammadi et al. (28) showed that in DU145 cells higher drug concentration decreased colony formation ability but the comet assay did not show a significant difference between the DNA damage in samples treated with the 50 µM concentration and the control group. The higher concentrations increased cell damage. The cells treated with 5-FU led to an arrest in the cell cycle in the S and G0/G1 phases. Choi and Ku (29) and Urick et al. (30) conducted the cell cycle test and colony formation assay. They reported that radiotherapy caused increased cellular absorption of 5-FU and the cells arrested at the G2/M and G1/S phases. This arrest was greater in the G2/M phase because of higher sensitivity to radiation. In addition, SSB, DSB, mitotic death, and apoptosis increased with radiation. Radiation activated the DNA damage signalling patway (11). These results indicated that 5-FU+radiation led to increased cytotoxicity and genotoxicity. Of note, the combined treatment of Mx with 5-FU could not increase toxicity and DNA damage in the cells. However, treatment with gamma radiation and 5-FU significantly increased cell damage. Treatment of irradiated cells with 5-FU+Mx showed that the combination of 5-FU+Mx significantly increased radiation-induced cell damage. Previously, Adamsen et al. (31) showed that 5-FU induced SSB followed by DSB in the HT29 cancer cell line. Bulgar et al. (32) showed that Mx markedly increased the comet assay detection of DNA damage due to fludarabine in human lymphoid malignancies. These studies showed that the increased tail moment by Mx and 5-FU measured by the alkaline comet assay might be caused by increased numbers of damaged DNA. This study confirmed the results of a study by Taverna et al. (13). They observed that the absorption of IUdR in the presence of radiation increased. The increased number of AP-sites led to the BER repair pathway in cells. Mx could increase cell damage by inhibiting the same repair pathway. 

## Conclusion

Our results showed that higher concentrations of 5-FU or Mx could increase cell toxicity and decrease cell proliferation ability. We showed that a low concentration of Mx did not significantly affect the level of cytotoxicity and DNA damage of 5-FU. In contrast, the low dose of gamma radiation significantly increased cytotoxicity and DNA damage by 5-FU. In the current study, Mx increased 5-FU induced radiosensitization of the HT29 colon cancer cell line. Therefore, our data suggested a new strategy for anticancer therapy for colon cancer. The combined Mx, 5-FU and gamma radiation treatment could increase cytotoxicity and genotoxicity compared to 5-FU and gamma radiation. Mx could be used as a modality to improve efficacy of 5-FU-based radiotherapy against colon cancer. 
